# A Call to Action: “Low-Dose Radiation May Help Cure COVID-19…” [Taps Mic] “…Is This Thing On?”

**DOI:** 10.1093/jncics/pkaa105

**Published:** 2020-11-19

**Authors:** Mohammad K Khan, Clayton B Hess

**Affiliations:** 1 Department of Radiation Oncology, Winship Cancer Institute, Emory University, Atlanta, GA, USA; 2 Department of Microbiology and Immunology, Emory University, Atlanta, GA, USA

The oldest habit in the world for resisting change is to complain that unless the remedy to the disease should be universally applied it should not be applied at all. But you must start somewhere. -Winston Churchill

In this issue of *JNCI Cancer Spectrum*, Venkatesulu et al. (1) provide a concise review and much-needed status update of low-dose radiotherapy (LD-RT) treatment for coronavirus disease 2019 (COVID-19). The authors step through the logic behind LD-RT and its promises and pitfalls. LD-RT can counter inflammation by various mechanisms demonstrated in preclinical models: adhesion and kinetics of peripheral blood mononuclear cells (2), lowered E-selectin (3,4), increased TGF-β1 (5,6), downregulated CCL20 release (7), reduced TNF-α production through apoptosis induction of peripheral blood mononuclear cells (3,8), reduced IL-1 production (9), reduced L-selectin expression (4), modulated MAP kinases and protein kinase B (10), reduced NF-κB (11), increased IL-10 production, and M1 to M2 phenotype conversion through iNOS pathway suppression (12-14). Although many investigational drugs may target 1 or more of these pathways (15,16), LD-RT may target multiple or all of them at once (17,18). The authors include mechanistic counterarguments: that LD-RT may activate and primate macrophages, which could worsen antiviral response, and reduce lymphocyte population with uncertain effect—detrimental or not (19,20).

## Where We Stand and Why

Currently, the only therapy that extends survival in COVID-19 is dexamethasone and still the death toll continues to climb. Additional therapeutics are needed. Parallel efforts to pursue new therapeutics and/or vaccines do not negate each other. If anything, arguing that drug development efforts render the study of LD-RT futile (21,22) reveals only naiveté for the urgency of life-and-death dramas that have played out more than 200 000 times within our own borders and a million times globally. The argument to not study LD-RT in humans also reveals a first-world–centric lack of awareness about global barriers to care where LD-RT may be a more accessible and cost-effective alternative compared with newer targeted drugs, likely to remain fiscally inaccessible across the world. Based on our RESCUE 1-19 experiences, a treatment capable of altering the course of COVID-19 that is already available in many global regions may be resting in our hands. Some have argued to let the opportunity pass, deferring to conventional drug therapies that may or may not materialize (21,22). Sure, we could pass by on the other side, but another will have to play Samaritan if we find ourselves standing at the plate and opt to not even swing the bat.

## Asking the Right Question

We last editorialized about LD-RT for COVID-19 in a May 2020 *ASCO Post*, drawing World War II-to-COVID era parallels and recalling that sulfathiazole saved Winston Churchill from streptococcus pneumonia (23). Together with the horrific memory of nuclear weapons, the dawn of the antibiotic era may have left infectious diseases—as an entire categorical entity—unexplored and forgotten as a potential therapeutic target for LD-RT. COVID-19 has now reinvigorated this debate. In what might be further pioneering work, we can think of nothing more relevant to add to the discussion at this time than insights into what it has been like to engage SARS-CoV-2 up close in personal combat these last months. Operationalizing the first trial of LD-RT for COVID-19–related acute respiratory distress syndrome has given us a lens from the front line that has convinced us (and our team of more than 130 volunteer staff and collaborating faculty) of the merits of this scientific pursuit. In this editorial, we aim not just to defend our clinical trial decisions but also to boldly and swiftly turn the tide of academic opinion by persuasive argument and reproducible data. We aim to refocus the entire radiation oncology community away from asking the question, “Should we be pursuing LD-RT to treat COVID-19?” and instead, sound a clarion call to action. Let us ask instead, “Who among us will rise to the occasion?” to speed the evaluation and validation of LD-RT as a COVID-19 treatment option with considerable global potential.

## Life Doesn’t Randomize, It Repeats

Most of what life teaches is through reproducibility not randomization. In the 1940s, observers related what to expect after LD-RT for infectious pneumonia:

A patient with a high fever, severe dyspnea, and cyanosis is irradiated. A few hours later, often within a period of six hours, he states that he can breathe more easily, and he takes some nourishment. After twelve to twenty-four hours the fever abates, in most cases by crisis, breathing is no longer painful, and dyspnea decreases or disappears entirely… indeed the whole course of the disease appear to have been definitely hastened by irradiation. And as this observation was made consistently, it would seem to be an established fact. (24)

At outset, knowing COVID-19 to be a distinctly separate entity than prior infectious pneumonias, and knowing the limitations of and need for controls within the cited observational data, we aimed to assess any hour-by-hour or day-to-day clinical response to LD-RT in patients with COVID-19. On April 24, 2020, two COVID-19 patients became the first in the modern era to receive LD-RT for an infectious indication. Both had COVID-19–related delirium and were nonverbal and dependent on oxygen. Both had been consented by proxy family members to undergo the experimental treatment based on historical and preclinical observations. Weeks of strategic planning had culminated in a dress rehearsal the night prior, and the treatment and infection prevention workflow went off perfectly. We transported the patients to the quarantined linear accelerator, caught our first glimpse of COVID consolidations on megavoltage imaging, treated, returned each to their hospital room, and waited. At hour 24, upon entering their hospital rooms, we were surprised. Both greeted us with open eyes and smiles having been weaned to room air. Both conversed about television or sports and had dramatic drops in inflammatory markers and joyful phone calls with reuniting family members. The 1940-era prediction of a clinical response to LD-RT was undeniably reproduced and therefore deserving of further study to determine causality vs coincidence. Such was our introductory experience to the role of LD-RT for human infection in the modern era.

## Transport, Treat, Repeat

It didn’t take long after treating our first patients for us to realize that C-reactive protein levels predictably fall the morning after LD-RT and over subsequent days in all but the sickest patients. It consistently and sometimes dramatically dropped like an inverted letter *V*. The question became, “Would it stay down?” If it did, de-escalation of oxygen requirement tended to follow. It also appears that the rapidity of decline could be dose- and disease-burden dependent, although this hypothesis requires further study. Analysis from our first 10 patients has been publicly released (25). Our experience substantiates the hour-by-hour trend for clinical improvement seen in the 1940s and that the mechanistic descriptions the authors described yield clinical results. Although randomization is needed to prove a causal relationship, reproducibility of statistically significant findings is highly informative and validates 1 irrefutable conclusion: LD-RT merits further study. We have now treated more than 40 patients and are evaluating our findings against another set of controls and a randomized trial that is part way through its planned accrual. Ameri et al. from Tehran, Iran, also treated 5 patients with LD-RT using 0.5 Gy and reported reductions in C-reactive protein beginning at day 1 in 4 of 5 patients, after we had released our initial data on preprint server (26). We saw the same in our first 5 patients treated with LD-RT (27). As more patients are treated, capacity for signal detection will only strengthen.

## Mouse Models: Demanding Small Instead of Standing Tall

The authors’ preclinical and clinical rationales support human clinical trials, yet they wisely warn that trials should carefully balance risk and benefit. Some have argued instead that “lack of supporting data makes the risks of a clinical trial of radiation therapy as a treatment for COVID-19 pneumonia unacceptable” (21,22) and have demanded the prioritization of animal models over clinical trials. Lab studies are certainly needed but must be balanced with the opportunity cost of foregoing human trials of considerable potential impact. Practical challenges and time requirements complicate the ability to generate SAR-CoV-2 mouse models in BSL3 labs. It is also unclear if the knowledge gained from mouse model experiments will ultimately have translatability to humans, which would thereafter require clinical trials in humans anyway, using a treatment we already know to be safe. Yet, armed only with a potential mechanism of adverse reaction and no substantiating data, some have called thoughtful consented study of LD-RT in humans unethical (21,22). These authors have cited outlying data points relating to second malignancy risk, which has maximized the perceived risk of LD-RT. Although we stand in support of capable basic and translational colleagues, on this occasion, we were disappointed by efforts that have skewed the scientific community’s perception of the risk-to-benefit ratio of LD-RT for COVID-19 so that it resembles little of the reality we see on the front line. We need go no further than to say that the recent memory of patients who died in our care argue against these claims. No COVID patient of ours nor of the readership, staring down the barrel of an impending endotracheal tube, should weigh a 1% and far distant second malignancy risk as equal to the pandemic’s approximate 50% intubated mortality and morbidity risk.

Therefore, we stand to champion the pivoting of our collective focus away from second cancer risk and toward the merits of the intervention itself. This is not a haphazard or shortsighted application of radiation like childhood tinea capitis (28), nor is it a resurgent misconception of LD-RT as a harmless cure-all as in days past (29). LD-RT carries carcinogenic risk, yes, but it appears to have a potentially large therapeutic effect against a much riskier COVID-19 when given after oxygen dependency but before respiratory failure. So let us be clear: LD-RT may prevent intubation (10% vs 40%) and hasten clinical recovery (3-fold improvement) (30). “Is anyone out there listening? Is this thing on?” The need to confirm these data is strikingly urgent. So why are we conjecturing about distant cancer risk when acute mortality is killing thousands each day? Intentional or not, the fear cultivating that now surrounds this debate has blinded many and continues to minimize the looming catastrophe that patients face. Indeed, COVID-19’s intubated 30-day mortality risk far exceeds that of any cancer.

## Conclusions

The debate over the role of LD-RT in infection has resurfaced ferociously and is likely only just beginning. Vast therapeutic potential remains untapped as “radiation immunology” may soon describe not only RT’s role in cancer immunology but also LD-RT’s role in benign immunopathologies. Therefore, LD-RT for COVID-19–related acute respiratory distress syndrome must be evaluated urgently, but it may just be the first of many infectious indications that await exploration. Collectively, we appear next up to bat on the global stage facing the pandemic to show what good LD-RT can do; let’s plant our feet and set our eyes on a homerun or base hit—anything but the inaction of a looking strikeout.

## Funding

None.

## Notes

**Role of the funder:** Not applicable.

**Disclosures:** Both authors disclose a preliminary provisional patent for the development of portable LD-RT equipment for COVID-19 treatment through commercial relationship with CureRays^TM^ Inc. The authors disclose a provisional patent through Emory University, the University of Tennessee at Knoxville, and CureRays^TM^ Inc.

**Author contributions:** MK and CH contributed equally to this editorial.

## Data Availability

Not applicable.
